# The Hungarian Adaptation, Validity and Reliability of the Questionnaire “Health Questionnaire on Back Care Knowledge in Daily Life Physical Activities for Adolescent Students” Examining the Back Care Knowledge and Spine Disease Prevention

**DOI:** 10.3390/jcm14082828

**Published:** 2025-04-19

**Authors:** Brigitta Szilágyi, Alexandra Makai, Borbála Magyar, Nóra Gulyás-Tanács, Gábor Rébék-Nagy, Klaudia Gál-Kiss, Péter Sándor Tardi, Zsófia Kovács-Szabó, Melinda Járomi, Nikolett Tumpek

**Affiliations:** 1 Doctoral School of Health Sciences, Faculty of Health Sciences, University of Pécs, 7621 Pécs, Hungary; bridzse@gmail.com (B.S.); gulyasnora.gyogytorna@gmail.com (N.G.-T.); zsofia.szabo@etk.pte.hu (Z.K.-S.); nikolett.tumpek@etk.pte.hu (N.T.); 2 Aurora Medical, 7624 Pécs, Hungary; 3 Faculty of Health Sciences, University of Pécs, 7621 Pécs, Hungary; magyar.b.borbala@gmail.com (B.M.); klau.kiss91@gmail.com (K.G.-K.); peter.tardi@etk.pte.hu (P.S.T.); jaromi@etk.pte.hu (M.J.); 4 Physical Activity Research Group, Szentágothai Research Centre, University of Pécs, 7622 Pécs, Hungary; 5 Department of Languages for Biomedical Purposes and Communication, Medical School, University of Pécs, 7622 Pécs, Hungary; gabor.n.rebek@aok.pte.hu

**Keywords:** child, back care knowledge and spine disease prevention, questionnaire, reliability, validity

## Abstract

**Background:** There is a small number of questionnaires for children in the international literature that assess back care knowledge and spine disease prevention. A back care knowledge questionnaire in Hungarian for 14–17-year-old children is not yet available. This study aimed to translate and adapt the back care knowledge questionnaire published by Monfort et al. into the Hungarian language and to examine its reliability and validity in assessing the back care knowledge of 14–17-year-old children. **Methods:** This cross-sectional study included 253 (134 girls and 119 boys) adolescents, with a mean age of 14.84 (14–17) years. The questionnaire adaptation was performed according to Beaton’s six-step principle. To test its internal consistency, the Kuder–Richardson 20 formula, containing binary variables, was used to assess the reliability of the questionnaire. The test–retest reliability was examined by the intraclass correlation coefficient (ICC). SPSS 27.0 software was used for data analysis, and the results were considered significant at *p* < 0.05. **Results:** The internal consistency measured by the Kuder–Richardson 20 coefficient examining the reliability of the questionnaire was 0.514. The test–retest reliability measured by intraclass correlation coefficients was 0.992 (0.985–0.996) *p* < 0.001. According to the Health Questionnaire on Back Care Knowledge and Spine Disease Prevention for 14–17-year-old children, the level of back care knowledge was 57.2%. **Conclusions:** The back care knowledge of Hungarian children is around 57.2%, which is lower than the data published in the international literature (60–70%). The Hungarian version of the questionnaire assessing the back care knowledge of 14–17-year-old children, the “Health Questionnaire on Back Care Knowledge and Spine Disease Prevention for 14–17 years old children (HEQBACK-14–17)”, was found to be a suitable back care knowledge measuring tool among 14–17-year-olds; however, the development or adaptation of more measurement tools is needed for better understanding and more precise examination.

## 1. Introduction

Nonspecific low back pain (nsLBP) is a significant public health issue and the most common musculoskeletal pain in adulthood. It severely impacts the patients’ quality of life and is the leading cause of adulthood disability worldwide. The number of nsLBP cases is also increasing among children, ranking as the fourth most common condition in individuals aged 10–14 years. The prevalence of nsLBP in children ranges from 13% to 51%, and its incidence increases with age. Back pain in children and adolescents can persist in adulthood. According to the literature, posture problems in childhood are appearing earlier and earlier, which can result in several unpleasant consequences; therefore, it is important to form good posture habits and acquire relevant back care knowledge in early childhood [1,2,3,4].

Enhancing disease-specific knowledge is crucial for disease prevention, as it improves rehabilitation effectiveness and prevents complications and recurrences. The importance of disease-specific education has been recognized in various conditions, such as diabetes, stroke, osteoarthritis, osteoporosis, and low back pain [5,6,7].

Increasing children’s back care knowledge and developing back care skills could significantly improve public health outcomes. Numerous studies have demonstrated that improving back care knowledge leads to better quality of life. Additionally, spine prevention knowledge plays a key role in fostering a spine-friendly lifestyle and healthy posture habits. However, children and adolescents generally have low levels of back care knowledge [2,3,8]. According to international surveys, the average back care knowledge of children aged 10–16 is 60–70%, while the average knowledge of adults is 35–57% [9,10].

School-age years represent a critical period for acquiring and automating health-related behaviors, spine-friendly movement patterns, and posture habits, integrating them into children’s behavioral routines. Promoting physical exercise, postural hygiene, physical activity, and spine prevention knowledge is essential. Individuals with accurate knowledge better understand processes affecting their health, can make better decisions regarding spinal prevention and rehabilitation, and participate more effectively in these processes [7,11,12].

The number and significance of back care education programs are increasing, yet there are only a few tools or questionnaires available to assess back care and spine prevention knowledge comprehensively. Back care knowledge is measured by questionnaires. The term comparability of international scientific results refers to the accessibility of the questionnaires in different languages [2,3,8,13].

In the international literature, Akbari-Chehrebargh et al. developed a tool consisting of 49 items, which examines spine-related behavior, behavioral capabilities, and back care knowledge of fifth-grade children, and is available in English [14]. Monfort et al. developed and validated the 24-item questionnaire for 14–17-year-old adolescents, which measures the anatomical, biomechanical, and ergonomical knowledge, and is available in English [15]. Miñana-Signes et al. developed a 13-item questionnaire addressing proper spine care and physical activity questions for 13–18-year-olds, available in both English and Spanish [16].

Four questionnaires assess cervical, back, and lumbar spine pain and its consequences in children [8,17,18,19]. Among these, the Young Spine Questionnaire has been validated in four languages and is available in nine languages.

In Hungary, there is only one validated questionnaire for children and one for adults related to back care knowledge and spine disease prevention. Szilágyi et al. developed and validated a 7-item back care knowledge questionnaire for 6–10-year-old children, which examines knowledge of spinal anatomy, biomechanics, and ergonomics [20]. The Low Back Pain Knowledge Questionnaire (LKQ) measures the back care knowledge and disease-specific knowledge of adults. The Hungarian adaptation was published by Kovács-Babocsay in 2019 [13].

In Hungary, several programs exist to promote spinal health knowledge or improve posture among children of different ages [21]. The Amazing Spinal Trip [22] is designed for 4–7-year-old children; “Porci Berci” targets children between the ages of 7–12 [23]; Posture Correction [24] and the Back School Program of Conscious Seating [25] aim to improve lumbar motor control and trunk muscle function for children. In certain programs, there are questions regarding back care knowledge, but they do not contain specific back care knowledge questionnaires. There is no known questionnaire validated in the Hungarian language measuring back care knowledge and spine disease prevention in 14–17-year-old children.

Monfort’s questionnaire, published in 2016, assesses the anatomical, biomechanical, and ergonomic knowledge of the spine in children aged 14–17. The other questionnaires for measuring children’s knowledge about the spine at this age group assess significantly less knowledge and do not cover as many areas as this questionnaire [15].

Research has addressed disease-specific knowledge across various age groups and health conditions. In Hungarian, validated questionnaires assessing back care knowledge are currently available for children aged 6–10 years [20] and for individuals aged 18 and above [13]. To enable the assessment of back care knowledge among adolescents aged 10–18, the present study employed this questionnaire for further development.

A cultural adaptation and validation process was undertaken to ensure the instrument’s suitability for this age group and to allow for international data comparison. The adapted tool enables the monitoring of age-related changes in back care knowledge and facilitates the evaluation of the effectiveness of back school and spinal education programs in both national and international contexts.

The aim of our study is to perform the Hungarian translation, adaptation, and validation of the questionnaire for 14–17-year-old adolescents published by Monfort et al. and to assess the back care knowledge of Hungarian adolescents between the ages of 14 and 17.

## 2. Materials and Methods

### 2.1. Study Design and Setting

The cross-sectional study population consisted of 253 participants (134 female and 119 male), with a mean age of 14.84 years (14–17). This study was conducted in educational institutions in Hungary between September 2020 and February 2021, using self-administered paper-and-pencil questionnaires. The sampling method was purposive non-randomized sampling. The selection was limited to schools and classes offering a general curriculum, without enhanced biology instruction or specialized programs (e.g., sports or dance arts) that could have provided students with above-average knowledge related to spinal anatomy, biomechanics, or ergonomics.

This study was conducted in line with the Declaration of Helsinki. This study was approved by the Institutional Review Board of the Regional Research Committee of the Clinical Center, Pécs, Hungary (case number 8444/PTE2020).

### 2.2. Eligibility and Selection of the Participants

Hungarian-speaking adolescents between the ages of 14 and 17 who met the inclusion criteria were invited to complete the questionnaire. Applicants with severe learning, reading, or speech recognition disorders were excluded [5,20].

The completion of the questionnaire was supported by the heads of institutions and children’s parents, who gave their written permission after reading the informed consent form provided to them before data collection.

### 2.3. Variables and Validation Procedure

Monfort et al. developed the Health Questionnaire on Back Care Knowledge in Daily Life Physical Activities for adolescent students at Valencia University, Spain. The questionnaire consists of 24 questions, containing 7 categories: (1) the topographical-anatomical category (Questions 1, 2, 3, and 6); (2) the functional-anatomical category (Questions 4, 5, and 7); (3) the standing posture category (Questions 8, 9, and 10); (4) the sitting posture category (Questions 11, 12, and 13); (5) the lying posture category (Questions 23 and 24); (6) the carrying weight in a schoolbag category (Questions 14, 15, 16, 17, and 18); (7) the lifting heavy objects category (Questions 19, 20, 21, and 22) [26].

In the Hungarian version of the questionnaire, the number of questions was 20. The categories and questions were structured as follows: (1) the topographical-anatomical category (Questions 1, 2, 3, and 6); (2) the functional-anatomical category (Questions 4, 5, and 7); (3) the standing posture category (Questions 8 and 9); (4) the sitting posture category (Questions 10, 11, and 12); (5) the lying posture category (Questions 19 and 20); (6) the carrying weight in a schoolbag category (Questions 13, 14, 15, and 16); (7) the lifting heavy objects category (Questions 17 and 18).

For each incorrect answer, the score was 0 points, while for each correct answer, the score was 1 point. The level of knowledge competence was measured by the total score, with values between 0 and 1, according to the authors’ suggestion [15]. Throughout the evaluation of the questionnaire, we also displayed the total results by percentage.

**Figure 1:**
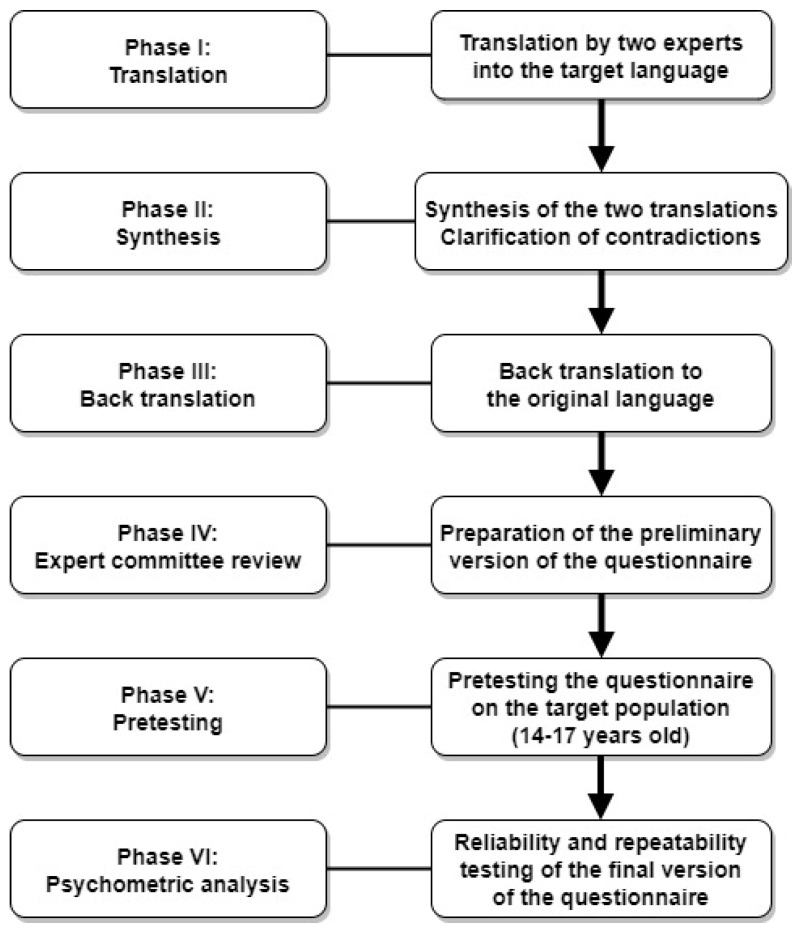
Phases in the translation and validation procedure.

The validation of the present questionnaire was conducted using Beaton’s 6-stage model: translation, synthesis, back-translation, expert committee review, pretesting, and examining the psychometric properties of the questionnaire [26,27] (Figure 1).

The translation, synthesis, and back-translation were completed by a four-member expert panel: a professional translator and physiotherapist, an English teacher, an employee of the Department of Languages for Biomedical Purposes and Communication, an MSc physiotherapist, and a Ph.D.-qualified physiotherapist. The opinions of all expert panel members were weighted equally, and the final formulation of the questionnaire items was determined through consensus based on their collective professional judgment.

### 2.4. Pretesting

We carried out the pretesting among 14–17-year-old adolescents. Some Hungarian versions of phrases were corrected in the course of pretesting: for the back was changed to for the spine; my feet are on the floor was changed to my feet are on the ground; sleeping position was changed to sleeping body posture. Considering the layout of the questionnaire, the letters indicating the optional answers were placed before the answer text.

### 2.5. Test–Retest

A total of 162 participants were involved in the test–retest process. There was a one-week interval between the first and second administration of the questionnaire. During this period, the children did not receive any classes or lessons containing spine-related content. The questionnaire requires specific domain knowledge, and no improvement was observed in the correct answers to either the simpler questions or the more complex items, where a lack of knowledge persisted across both measurements.

### 2.6. Psychometric Analysis

#### 2.6.1. Sample Size Estimation

The planned sample size in our research was 240 adolescents, with the final sample consisting of 253 participants, exceeding the initial plan based on the suggested ratio of 10 participants per item in the literature for validation procedures.

#### 2.6.2. Statistical Analyses

Descriptive statistics were presented as mean and standard deviation (SD) or frequency and proportion (%) for participants’ characteristics variables and for measured outcomes.

The reliability of the final 20-item questionnaire was examined using the Kuder–Richardson 20 (KR-20) formula; according to the formula, the examined tool can be considered reliable above the value of 0.5 [28,29]. The repeatability test was examined by the test–retest method with the intraclass correlation coefficient (ICC). The value of ICC referred to a weak correlation under 0.40, a moderate correlation under 0.75, and a strong correlation above 0.75 [20]. We performed statistical calculations using SPSS 27.0 (IBM SPSS, IBM Corp, Armonk, NY, USA) software. The results were considered significant at *p* < 0.05.

## 3. Results

### 3.1. Descriptive Data

In our cross-sectional validation study, 253 participants were involved. The majority of participants (54.9%) were female, with the most common age group being 15 years (41.4%). Notably, 71% of the individuals reported engaging in regular sports activity, while 9.9% had a diagnosed spine disease (Table 1).

### 3.2. Results of the Validity and Reliability of the Questionnaire

As a first result, examining the reliability of the 24-item questionnaire, the value of the KR-20 coefficient was 0.449. Based on the results indicating low internal consistency, the committee decided to exclude four questions; thus, Questions 9, 14, 19, and 22 were deleted from the questionnaire. The ninth question was about choosing the proper posture in a schematic illustration, but even the correct interpretation of the illustration often caused problems. The 14th and 19th questions included the option of carrying a load in a rolling bag among the possible answers, which is not a widely known and used type of bag among Hungarian students at present. The 22nd question examined the method of overhead lifting, which is more related to accident prevention than to spinal health (Table 2).

In the course of evaluating the reliability of the final 20-item questionnaire, the value of the KR-20 coefficient was 0.514. We can state that the 20-item questionnaire is reliable/the reliability of the questionnaire is proven. The repeatability of the questionnaire, assessed through a test–retest procedure, yielded a total correlation coefficient (ICC) of 0.992 (0.985–0.996, *p* < 0.001). Regarding the seven categories, the intraclass correlation coefficient between test and retest for the topographical–anatomical category was 0.955 (0.917–0.976, *p* < 0.001); for the functional–anatomical category, it was 0.989 (0.980–0.994, *p* < 0.001); for the standing posture category, it was 1.00; for the sitting posture category, it was 0.990 (0.982–0.995, *p* < 0.001); for the lying posture category, it was 0.970 (0.945–0.984, *p* < 0.001); for the carrying weight in a schoolbag category, it was 1.00; and for the lifting heavy objects category, it was 0.961 (0.928–0.979, *p* < 0.001). According to the test–retest examination, the questionnaire is a reliable tool for measuring back care knowledge.

According to the results, the level of back care knowledge is 57.2%. By examining the different categories of the questionnaire, the topographical–anatomical knowledge performance was 61.1%; the functional–anatomical knowledge performance was 76.9%; knowledge performance on the standing posture question was 68.1%; on the sitting posture question, it was 51.8%; on the lying posture, it was 29.3%; on the carrying weight in a schoolbag question, it was 61.3%; and on the lifting heavy objects question, it was 52.1% (Table 3). A total of 13 questions were answered correctly by 50% or more of the respondents, and seven questions were answered correctly by less than half of the respondents (Table 4).

## 4. Discussion

The literature contains only a limited number of questionnaires designed to measure children’s back care knowledge. Among these, some are not validated. Validated questionnaires are typically suitable for assessing back care knowledge in children aged 6–18 years (Table 5). However, cultural adaptation and validation of these questionnaires are available in only a few languages. In Hungarian, there is a validated questionnaire only for the 6–10 age group. The aim of this study was to adapt and validate a knowledge assessment questionnaire specifically for Hungarian-speaking adolescents aged 14–17 years [2,10,16,30,31,32].

We assessed the back care knowledge of the 14–17-year-old Hungarian children with a shorter form of the original questionnaire published by Monfort et al. During Beaton’s six-stage validation model, after the approval of the committee, the original 24-item questionnaire became a 20-item questionnaire in the Hungarian version, as a result of which the number of categories did not change, with only the number of questions within each category being adjusted. The scores of the categories and questions of the questionnaire are not published in the article by Monfort et al., and the questionnaire has not been validated for other languages; thus, these results cannot be compared (Table 6).

The Hungarian children’s back care knowledge scores are lower than those of the Spanish children. We could find the largest differences in the first, fifth, and seventh categories regarding topographical–anatomical knowledge, lying posture, and carrying weight in a school bag. In the third, fourth, and sixth categories, which included the questions regarding standing posture, sitting posture, and carrying weight in a schoolbag, we found similar results. In both studies, the children achieved the lowest scores in the category concerning lying posture, proper sleeping position, and optimal mattress firmness (Table 7). The 0.514 reliability coefficient that resulted from this 20-item questionnaire supports the above-mentioned results and is acceptable but lower than the 0.820 value published by the original authors. In addition, four questions that were hardly interpretable/intelligible in Hungarian culture further weakened the value.

The reliability coefficient is significantly affected if the questions of the measuring tool prove to be difficult for the participants, which is also confirmed by the scores representing the level of knowledge. The results of the repeatability test also refer to the difficulty of the questions because the results of the test–retest stage showed a strong correlation.

The questionnaire is relatively rare and unique. No other validation studies were conducted with the questionnaire, so we could not compare the results. Therefore, we compared the values of the change in knowledge level with the results of other international research articles. Accordingly, the discussion section has been supplemented to reflect this comparison by knowledge levels (Table 8).

To date, no additional studies have been conducted on the reliability of HEQBACK-14–17. Consequently, the psychometric properties of the Hungarian version of this questionnaire (GEPT-14–17) cannot currently be compared with other versions.

Validated back care knowledge measurement tools and questionnaires have been available for children since 2015. Prior to this, children’s knowledge was assessed using non-validated, self-developed questionnaires. Currently, validated back care questionnaires for children are available in only a few languages, in contrast to those assessing children’s spinal pain or adult back care knowledge. For adults, the back care knowledge questionnaire has been validated in seven languages. Back care questionnaires designed for different age groups of children are available for ages 6 to 18. Children’s back care knowledge is generally very low, increases with age, but remains relatively low overall. Back care education programs significantly improve children’s knowledge of spinal disease prevention [2,7,8,11,16,31].

The results obtained from the questionnaire show which groups of questions adolescents have less knowledge in, which areas need to be developed, e.g., spinal anatomy, spinal biomechanics, ergonomics, correct posture (sitting and standing), correct lying position, correct lifting, and correct weight bearing.

The results obtained from the questionnaire help inform the development of age-appropriate standard educational programs and the individualization of the programs. There are no precisely defined spinal educational programs standardized for age. The main focus areas of spinal education programs are known, such as spinal anatomy (bone, musculature), biomechanics (biomechanical properties of motion segments, spinal movements and their biomechanical effects), and ergonomic knowledge (correct posture, working position, and posture while learning), rules for spinal protection and elements of a spine-friendly lifestyle, spine-friendly leisure, spine-friendly sports, development of spinal diseases, causes of spinal pain, prevention and treatment of spinal diseases. The content of the general spine education programs can be adaptable to individuals or groups based on the results, allowing for more effective education.

### Study Limitations

A larger proportion of participants in the questionnaire were aged 14–15 years, with fewer 16-year-olds, and no data were collected on 17-year-olds. Additionally, most participants were from county seats, resulting in limited information on the back care knowledge of children living in smaller towns or rural areas.

## 5. Conclusions

The Hungarian version of the questionnaire “Gerinchasználattal és –prevencióval kapcsolatos tudást felmérő kérdőív 14–17 éves gyerekek számára” (GEPT-14–17), “Health Questionnaire on Back Care Knowledge and Spine Disease Prevention for 14–17 years old children” (HEQBACK-14–17), assessing the back care knowledge of 14–17-year-old children, was found to be a suitable tool for measuring back care knowledge among 14–17-year-olds. The back care knowledge of Hungarian children is lower than the levels reported in the international literature. Hungarian children’s back care knowledge can be developed through back school programs.

The GEPT-14–17 questionnaire can be used to assess back care knowledge in Hungarian-speaking adolescents aged 14–17. This tool provides valuable insight into the effectiveness of interventions aimed at improving back care knowledge and promoting spine health in this age group.

The practical application of the questionnaire in healthcare lies in the prevention of spinal diseases in children and adults. Spinal pain occurs more and more frequently in childhood and at an earlier age. According to surveys, spinal diseases and pain in childhood predict the appearance of chronic nonspecific low back pain in adulthood. Appropriate spinal prevention knowledge helps prevent spinal pain and enables more effective rehabilitation, and reduces the number of relapses.

There is also a practical application of the questionnaire in education, since the effectiveness of spinal prevention educational and back school programs taking place within the school framework can be measured using the questionnaire.

In the future, it would be worth conducting the survey among rural populations and older adolescents. Furthermore, children’s back care knowledge should be compared with their academic performance. It could also be interesting to examine the parents’ back care knowledge and compare it with their children’s, or compare it with their education level.

## Figures and Tables

**Table 1:** Main characteristics of the sample (N = 253).

Main Characteristics		n	%
Gender	Male	114	45.1%
	Female	139	54.9%
Age	14 years	92	36.4%
	15 years	105	41.4%
	16 years	56	22.2%
Regular sports activity	No	73	29.0%
	Yes	180	71.0%
Place of living	County seat	114	45.1%
	City	72	28.4%
	Village	67	26.5%
Diagnosed spine disease	No	228	90.1%
	Yes	25	9.9%

**Table 2:** Health questionnaire on back care knowledge in daily life physical activities for adolescent students (HEBACAKNOW), Hungarian version, phase in the translation and validation process.

Phases I and II: Translation and Cross-Cultural Adaptation, Synthesis
During the translation of the questionnaire from English to Hungarian, no significant issues were identified. There were no concepts or expressions that were either incomprehensible or unused in Hungarian. Although there were differences between the two Hungarian translations, none of them altered the meaning of the sentences. Since the questionnaire is intended for young people, we aimed to use language that resonates with a youthful tone during both the translation and the design of the questionnaire.
**Phase III: Back-Translation**
During the back-translation review, no significant differences were observed between the back-translated and the original questionnaire. None of the translators reported notable difficulties during the translation process. The content of the original questionnaire and the back-translated version is consistent.
**Phase IV: Expert Committee Review**
The preliminary version of the questionnaire was developed and approved by the committee.
**Phase V: Pretesting**
The ambiguous expressions identified by respondents, which were subsequently revised, include: “for the back” replaced with “for the spine,” “my feet are on the floor” replaced with “my feet are on the ground,” and “sleeping position”, replaced with “sleeping body posture.” In terms of questionnaire layout, the letters indicating the optional answers were placed before the response options. Professionally debated terms raised during pretesting: The names of muscles in the questionnaire are also presented in Latin (Question 6). Based on their age, children at this stage of education are typically unfamiliar with Latin terminology. However, an exception was noted in the case of the term “biceps”, which is more widely recognized than its Hungarian equivalent, “kétfejű.” Consequently, the committee decided to include both terms in the questionnaire. Responding children reported challenges in understanding the posture illustrations in Question 9. At this age, children are rarely exposed to such representations of body postures. In Questions 12 and 14, the terms “book holder” and “rolling bag” refer to tools that are less common and less frequently used among children in Hungary. Following the pretesting phase, the committee reviewed and approved the final Hungarian version of the questionnaire.
**Phase VI: Psychometric Analysis**
Due to statistical considerations, only 20 out of the original 24 questions were retained, and the resulting questionnaire achieved a Kuder–Richardson 20 reliability coefficient of 0.514—an acceptable value, though notably lower than the 0.820 reported by the original author. This discrepancy is largely attributed to four questions that were nearly unintelligible within Hungarian culture, thereby diminishing the overall reliability, which was also influenced by the inherent difficulty of the items, as reflected in participants’ knowledge scores. Additionally, the strong test–retest correlation and an intraclass correlation of 0.992 (95% CI: 0.985–0.996, *p* < 0.001) further support the tool’s reliability. Overall, the Hungarian version of the questionnaire—referred to as the GEPT-14–17 or HEQBACK-14–17—was validated as a suitable instrument for assessing back care knowledge among 14- to 17-year-olds.

**Table 3:** Results of back care knowledge and spine disease prevention assessment among 14–17-year-old children.

24-Item Questionnaire	Mean	Median	SD	IQR 95% Lower	IQR 95% Upper
Topographical–anatomical category	0.61	0.50	0.17	0.50	0.75
Functional–anatomical category	0.76	0.67	0.26	0.67	1.00
Standing posture category	0.68	0.67	0.25	0.67	0.33
Sitting posture category	0.51	0.67	0.27	0.33	0.67
Lying posture category	0.29	0.25	0.32	0.00	0.50
Carrying weight in a schoolbag category	0.61	0.60	0.22	0.60	0.80
Lifting heavy objects category	0.52	0.50	0.22	0.50	0.75
Total score of knowledge (24 questions)	0.57	0.59	0.11	0.49	0.65
20-item questionnaire					
Topographical–anatomical category	0.61	0.17	0.50	0.50	0.75
Functional–anatomical category	0.77	0.26	0.67	0.67	1.00
Standing posture category	0.66	0.32	0.50	0.50	1.00
Sitting posture category	0.52	0.27	0.67	0.33	0.67
Lying posture category	0.29	0.32	0.25	0.00	0.50
Carrying weight in a schoolbag category	0.58	0.25	0.50	0.50	0.75
Lifting heavy objects category	0.79	0.30	1.00	0.50	1.00
Total score of knowledge (20 questions)	0.61	0.13	0.60	0.50	0.70

**Table 4:** Frequencies of the correct answers on the 20-item measurement tool.

Items	N	%
1. The spine is located in:	251	99
2. How many curves does the spine have?	34	14
3. How are the different parts of the spine called?	225	89
4. The spine has got curves in order to:	161	64
5. What is the function of the spine in the body?	186	73
6. Which of the following muscles is a trunk muscle?	108	43
7. The function of the trunk musculature is:	237	94
8. The most stressful posture for your back is:	111	44
9. When standing for a while without moving, I should:	222	88
10. When sitting during a long time (watching TV, studying, working, etc.), I should:	137	54
11. When sitting by a desk:	116	46
12. When sitting by a desk with a computer:	141	56
13. When carrying a schoolbag with books, the weight should be:	83	33
14. When carrying weight in my schoolbag, I should wear it on my back:	175	69
15. When carrying weight in my schoolbag:	183	72
16. When carrying weight in bags, I should:	150	59
17. When holding heavy loads in your arms, it is better to:	206	81
18. When lifting heavy objects off of the floor, I should:	194	77
19. When sleeping, the best posture is:	45	18
20. The surface where I sleep on should be:	103	41

**Table 5:** Back care and low back pain prevention knowledge questionnaires.

Questionnaire Name	Author	Language	The Main Groups of Questions	Item, Score	Age
Health Questionnaire on Back Care Knowledge and Spine Disease Prevention for 6–10 years old children (HEQBACK-6–10)	Szilagyi et al. (2021) [20]	English Hungarian	spine anatomy, spine biomechanics, ergonomic knowledge	7 items, total score 26 points	6–10 years
Health questionnaire on back care knowledge concerning practice physical activity and exercise for adolescents (HEBACAKNOW-PAE)	Miñana-Signes et al. (2015) [16]	Spanish English	back care, physical activity, physical abilities, posture	13 items, total score 10 points	13–18 years
Health questionnaire on back care knowledge in daily life physical activities (HEBACAKNOW)	Monfort-Panego et al. (2016) [15]	English	topographical spine anatomy, functional anatomy, standing posture, sitting posture, lying posture, carrying loads, lifting heavy objects	24 items	14–17 years
The Back-care Behavior Assessment Questionnaire (BABAQ)	Akbari-Chehrehbargh et al. (2020) [14]	English	spine-related behavior, behavioral capability (skills and knowledge), self-efficacy, expectation beliefs, performance spine	5 items, 49 questions, total score 132 points	5th-grade
Low Back Pain Knowledge Questionnaire (LKQ)	Maciel et al. (2009) [6] Kanaan et al. (2022) [32] Kovács-Babocsay et al. (2019) [13] Chawla et al. (2023) [7]	Brazilian Portuguese, English, Arabic, Hungarian, Hindi	General knowledge about the spine: spine anatomy, causes of low back pain, symptoms, diagnosis, prognosis, concepts related to spine diseases, therapy used in spine patients	16 items, total score 24 points	over 18

**Table 6:** Back care knowledge scores by category in the questionnaire.

Category	Questions	Minimum	Maximum	Mean	SD
Topographical–anatomical category	Question 1, 2, 3, 6	1.00	4.00	2.44	0.68
Functional–anatomical category	Question 4, 5, 7	0.00	3.00	2.30	0.76
Standing posture category	Question 8, 9	0.00	2.00	1.31	0.63
Sitting posture category	Question 10, 11, 12	0.00	3.00	1.55	0.80
Lying posture category	Question 19, 20	0.00	1.00	0.58	0.64
Carrying weight in a schoolbag category	Question 13, 14, 15, 16	0.00	4.00	2.33	1.00
Lifting heavy objects category	Question 17, 18	0.00	2.00	1.58	0.60

**Table 7:** Comparison of the back care knowledge and spine disease prevention among 14–17 years old children.

Studies (Median Values)	N	S1	S2	S3	S4	S5	S6	S7
Monfort et al. (2016) Spain [15]	230 students 15.23 (14–17) years	0.75	1.00	0.67	0.67	0.5	0.60	0.75
Present study, Hungary (20-item questionnaire)	253 students 14.84 (14–17) years	0.50	0.67	0.50	0.67	0.25	0.50	1.00

Abbreviations: S1–S7 = 7 subscales of the GEPT-14–17 questionnaire.

**Table 8:** Comparison of back care knowledge levels with international research.

Author (Year)	Age (Years)	Questionnaire	Total Scores of Back Care Knowledge (Points)	
			Pre-Intervention	Post-Intervention
Foltran et al., 2011 [30]	9–16	Back care questionnaire	3.6 ± 2.9	7.5 ± 2.2
Miñana-S et al., 2019 [33]	11.19 ± 0.4	HEBACAKNOW-PAE HEBACAKNOW-DL	2.36 ± 0.72 3.32 ± 1.24	6.56 ± 1.28 6.32 ± 1.57
Geldhof et al., 2006 [34]	11.3 ± 0.8	Back posture knowledge questionnaire	General back posture knowledge: 1.0 ± 3.9 Specific back posture knowledge: 4.9 ± 7.4	General back posture knowledge: 5.1 ± 2.9 Specific back posture knowledge: 7.5 ± 4.6
Habybabady et al., 2012 [31]	10–11	Questionnaire assessing knowledge and behavior	Knowledge: 43.4 ± 12.93 Behavior: 53.3 ± 16.34	Knowledge: 60.5 ± 24.32 Behavior: 65.5 ± 20.34
Dullien et al., 2018 [10]	10.59 ± 0.44	Knowledge test	14.42 ± 3.03	17.17 ± 2.84
Cardon et al., 2000 [35]	10.02	Knowledge test	−0.9	3.38
Szilágyi et al., 2021 [20]	6–7	HEQBACK-6–10	3.269 ± 3.341	16.269 ± 2.426

## Data Availability

The data presented in this study are available on reasonable request from the corresponding author. The data are not publicly available due to privacy restrictions.
